# Mendelian randomization analyses support causal relationships between systemic lupus erythematosus and brain imaging-derived phenotypes

**DOI:** 10.3389/fneur.2024.1444885

**Published:** 2024-11-22

**Authors:** Yan Ma, Rui Li, Qianqian Li, Wanyi Lin, Liangjing Lu

**Affiliations:** Department of Rheumatology, Renji Hospital, School of Medicine, Shanghai Jiao Tong University, Shanghai, China

**Keywords:** Mendelian randomization, neuropsychiatric systemic lupus erythematosus, systemic lupus erythematosus, neuroimaging phenotypes, brain IDPs, MRI

## Abstract

**Background:**

Neuropsychiatric disorders in systemic lupus erythematosus (NPSLE) are often accompanied by alterations in brain structure and function. Subtle changes in brain structure also can be observed in non-NPSLE patients. MRI can be used as a non-invasive tool to determine nervous system involvement in SLE. However, the causal relationship between SLE and brain MRI remains unclear.

**Methods:**

We designed two-sample MR analyses to identify brain IDPs associated with SLE. The GWAS summary data of 3,935 IDPs from the UK Biobank were used as outcomes in MR analyses.

**Results:**

There were 25 statistically significant causal relationships between SLE and brain IDPs, in which the several cortical area, anterior corona radiata, and posterior limb of internal capsule were included. These results may suggest the pathogenesis of neuropsychiatric symptoms in patients with SLE.

**Conclusion:**

The findings revealed strong genetic evidence for causal links between SLE and neuroimaging phenotypes. Our results provide a promising method for the daily assessment and monitoring of SLE patients.

## Introduction

The neuropsychiatric involvement in systemic lupus erythematosus (SLE) is a challenge for clinicians, both at a diagnostic and therapeutic level (1). Although the survival and prognosis of SLE have improved substantially in recent decades, neuropsychiatric lupus (NPSLE) continues to provide significant morbidity and mortality, only surpassed by lupus nephritis (2). Central NPSLE includes diffuse psychiatric or neuropsychiatric syndromes (diffuse NPSLE) and focal neurological syndromes (focal NPSLE). Cognitive impairment (present in up to 80% of cases), mood disorders (present in up to 80% of cases) and anxiety disorders (present in up to 40% of cases) are the most common symptoms of diffuse NPSLE, whereas headache (present in up to 28% of cases), seizures (present in up to 20% of cases) and cerebrovascular disorders (present in up to 15% of cases) are the most common focal NPSLE manifestations (3). Vasculitis, noninflammatory vasculopathy, and antiphospholipid (aPL) syndrome (APS) are involved in the pathogenesis of NPSLE (4–6). NPSLE remains difficult to diagnose due to the lack of validated biomarkers that can be accurately attributed to the clinical manifestations of NPSLE.

Neuroimaging can be used as a non-invasive tool to determine central nervous system involvement in SLE. MRI, which is more sensitive than CT and avoids radiation exposure, can be used to detect intracranial abnormalities and assess chronicity and evolution of these abnormalities (7). However, conventional MRI depicts abnormalities in only about 75% of patients with NPSLE (8). When conventional MRI results are negative, advanced neuroimaging such as magnetic resonance spectroscopy, perfusion-weighted MRI, or diffusion tensor imaging may be performed to detect occult imaging abnormalities. Moreover, non-NPSLE has been confirmed to have subtle changes in brain structure before the appearance of obvious neuropsychiatric symptoms (9). A few number of observational case-control studies have been performed to explore the relationships between brain imaging and SLE. For example, diffusion tensor imaging reveals brain white matter microstructural alterations associated with systemic lupus erythematosus. Compared to healthy controls, SLE patients had significantly lower fractional anisotropy (FA) values, and significantly higher mean diffusivity (MD), axial diffusivity (AD), radial diffusivity (RD) values in many white matter tracts (10). Different cerebral blood flow within gray and white matter were also observed between NPSLE and non-NPSLE patients (11). There is growing evidence that SLE is associated with alterations in brain imaging, but traditional observational studies are more likely to be influenced by residual confounders. Thus the causal relationship between brain imaging and SLE remains unclear.

Mendelian randomization (MR) is a useful genetic epidemiology study design using genetic variants as instrumental variables (IVs) to investigate whether the exposure is causally related to a medically relevant disease risk (12). Large-scale genome-wide association studies (GWASs) provide the opportunity to systematically explore the potential causal relationships between numerous brain imaging-derived phenotypes (IDPs) and SLE through MR (13). Accordingly, we designed a two-sample MR analyses to identify brain IDPs associated with SLE.

## Methods

**Figure 1:**
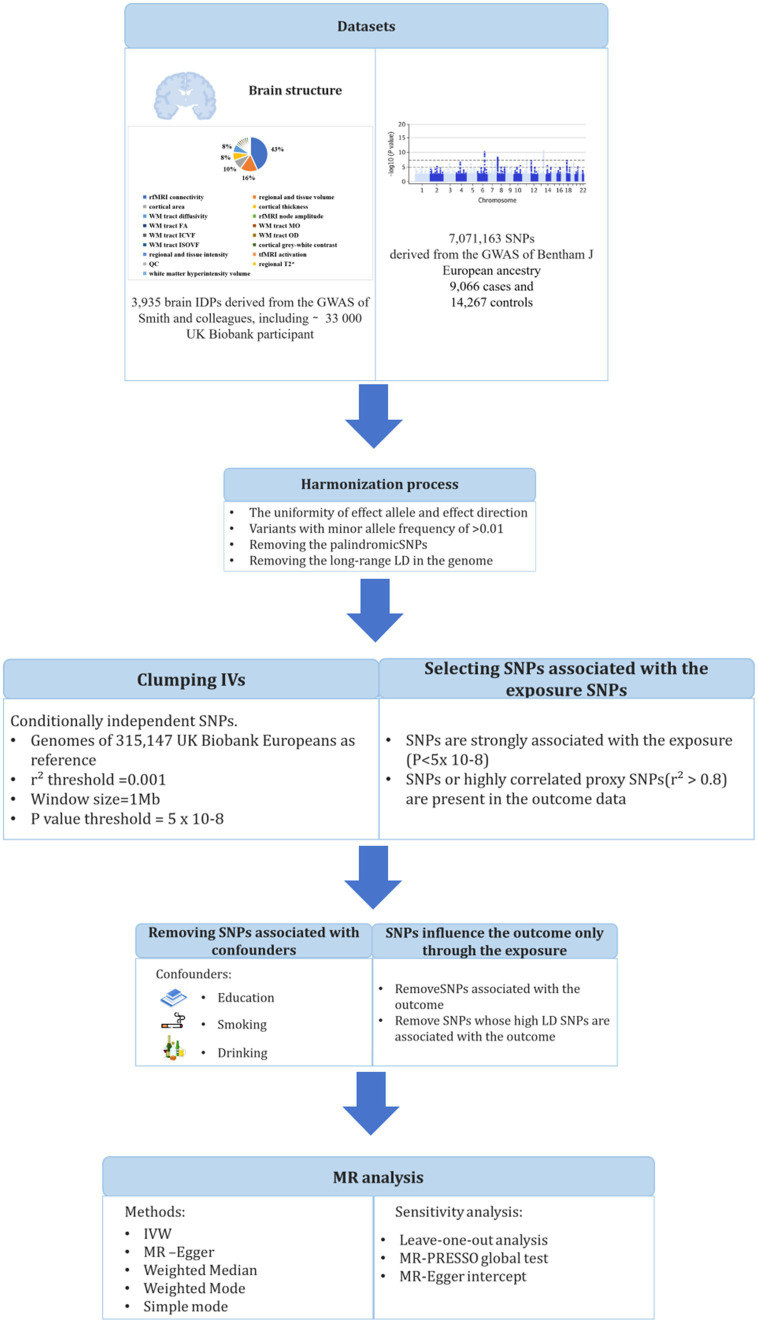
Workflow of the causal inference between systemic lupus erythematosus and brain imaging-derived phenotypes.

The study design is shown in Figure 1. We obtained the GWAS summary data of 3,935 IDPs from UK Biobank and used as an outcome in MR analyses (Supplementary Table S2) (13). Brain IDPs were named according to the original nomenclature (13). GWAS summary statistics of SLE was also obtained from the dataset processed by Bentham et al. (14). Ethical approvals were obtained by each individual participating cohort; therefore, no additional ethical approvals or informed consents were required. Then, we conducted two-sample MR using TwoSampleMR. There are three core assumptions of MR analysis: relevance, independence, and exclusion. (1) Relevance: the variables selected as genetic instruments are closely associated with exposures; (2) independence: genetic variation is not associated with confounding factors; and (3) exclusion restrictions: genetic variation affects outcomes only through exposures instead of other pathways.

### Selection of instrumental variables

In this study, genetic variants in SLE were extracted from a large GWAS study that included 5,201 cases of European ancestry and 9,066 controls of European ancestry (GWAS ID: ebi-a-GCST003156) (14). In order to ensure complete random independence of instrumental variable estimation and exclude the impact of linkage disequilibrium (LD) on outcome factors, a threshold (*p* < 5 × 10^−8^) was set to screen SNPs closely related to exposure factors in a genome-wide sense. Second, the minor allele frequency (MAF) threshold of the variants of interest was 0.01. Third, one of the principles of the MR approach is that there is no linkage disequilibrium (LD) among the included IVs, as the presence of strong LD might result in biased results. We set an LD pruning *R*^2^ threshold of 0.001 and a window size of 1 Mb for preprocessing to maintain consistency between the exposure effect allele and the outcome effect magnitude. The selection of IVs should satisfy the assumptions that SNPs are strongly associated with exposure but not with outcome and not with confounding factors. We considered three potential confounders that interfere with the relationship between brain structure and SLE, including education, alcohol consumption, and smoking behavior, which have been reported to affect brain structure in previous studies (15–17). We screened all IVs against the GWAS Catalog^1^ to assess any associations with potential confounders in European participants. Next, the *F*-statistic was calculated to estimate overlapping effects and weak instrumental bias, and *F* < 10 was considered a suspicious bias and excluded.

### Brain IDPs

Brain IDPs were obtained from the GWAS summary data processed by Smith et al., including 33,224 UK Biobank participants. Details of all neuroimaging measures and processing methods are provided by the online reference of UK Biobank.^2^ All GWAS summary statistics on IDPs were obtained from the Oxford Brain Imaging Genetics (BIG40) web browser.^3^ To fully reveal the effects of SLE on neurobiological structure and function, we included all brain IDPs for analysis.

### Statistical analysis

This two-sample MR analysis was performed using R software, the inverse variance weighted (IVW) method was used as the main analysis to evaluate the causal relationship between SLE and IDPs. To avoid the inflation of false-positive findings, we calculated the false-discovery rate (FDR) adjusted *p*-values across the IVW analyses. In addition, we supplemented IVW MR with MR-Egger, weighted median, weighted mode, and simple mode estimators, which rely on different assumptions than IVW as auxiliary analysis methods. Mendelian randomization pleiotropy residual sum and outlier (MR-PRESSO), left one sensitivity analysis, and Cochran’s *Q*-test were used to extensively evaluate MR analysis results. The MR-PRESSO test was used to examine the potential skewness of horizontal pleiotropic by identifying and excluding pleiotropic SNPs with *p* < 0.05 to reassess causal relationships. The leave-one-out method was used to determine the robustness of MR results by excluding SNPs. Cochran’s *Q*-test determined the heterogeneity leading to MR results based on IVW and MR Egger estimates.

Our study followed the Strengthening the Reporting of Genetic Association Studies (STREGA) reporting guideline and Strengthening the Reporting of Observational Studies in Epidemiology Using Mendelian Randomization (STROBE-MR) reporting guidelines.

## Results

### Forward MR of SLE on IDPs

We conducted a two-sample MR study, which was mainly using the IVW method to analyze, and FDR adjusted *p*-values <=0.05 was considered significant. We excluded some associations which the directions of the estimates from the other four MR methods were the different with those of the IVW method. There were a total of 25 statistically significant causal relationships. We discussed the results below according to the UK Biobank IDP categories.

### Cortical area

As shown in Table 1. For the white surface of right hemisphere parcellated by Destrieux (a2009s) parcellation, SLE associated with area of G + S-occipital-inf (*β* = 0.013, FDR-adjusted *p* = 0.050). In left hemisphere, SLE associated with isthmus of cingulate gyrus (*β* = 0.011, FDR-adjusted *p* = 0.049), area of S-pericallosal (*β* = 0.010, FDR-adjusted *p* = 0.048) and posterior cingulate (*β* = 0.010, FDR-adjusted *p* = 0.048).

**Table 1:** MR results of causal effects between systemic lupus erythematosus and imaging-derived phenotypes (IDPs) in cortical area.

IDPs (outcome)	Description	Number of SNPs	*β* (IVW)	SE (IVW)	*p*-value (IVW)	*p*-FDR
aparc-a2009s_rh_area_G + S-occipital-inf	Area of G + S-occipital-inf in the right hemisphere generated by parcellation of the white surface using Destrieux (a2009s) parcellation	42	0.013	0.005	0.015	0.050
aparc-pial_lh_area_isthmuscingulate	Area of isthmus cingulate in the left hemisphere generated by parcellation of the pial surface using Desikan-Killiany parcellation	40	0.011	0.006	0.040	0.049
aparc-a2009s_lh_area_S-pericallosal	Area of S-pericallosal in the left hemisphere generated by parcellation of the white surface using Destrieux (a2009s) parcellation	42	0.010	0.005	0.023	0.048
aparc-DKTatlas_lh_area_posteriorcingulate	Area of posteriorcingulate in the left hemisphere generated by parcellation of the white surface using DKT parcellation	42	0.010	0.005	0.029	0.048

### Regional and tissue volume

As shown in Table 2. In left hemisphere, there was significant causal association between SLE and the volume of pars triangularis (*β* = 0.012, FDR-adjusted *p* = 0.049) generated by parcellation of the white surface using Desikan-Killiany parcellation. SLE also had a significant effect on volume of pulvinar anterior (PuA) (*β* = 0.009, FDR-adjusted = 0.049) in the left hemisphere.

**Table 2:** MR results of causal effects between systemic lupus erythematosus and imaging-derived phenotypes (IDPs) in regional and tissue volume.

IDP category	IDPs (outcome)	Description	Number of SNPs	*β* (IVW)	SE (IVW)	*p*-value (IVW)	*p*-FDR
Regional and tissue volume	aparc-Desikan_lh_volume_parstriangularis	Volume of parstriangularis in the left hemisphere generated by parcellation of the white surface using Desikan-Killiany parcellation Volume of PuA in the left hemisphere	40	0.012	0.006	0.039	0.049
	ThalamNuclei_lh_volume_PuA	Generated by subcortical volumetric sub-segmentation of the thalamic nuclei	42	0.009	0.005	0.049	0.049

### rfMRI connectivity

Significant causal associations of IVW estimates were observed between SLE and 9 resting-state functional MRI (rfMRI) connectivity IDPs, accounting for 26% of all positive results, as detailed in Table 3. The significant rfMRI connectivity IDPs predominantly distributed in the ICA100 edge 600–800, accounting for about 45% of the total.

**Table 3:** MR results of causal effects between systemic lupus erythematosus and imaging-derived phenotypes (IDPs) in rfMRI connectivity.

IDP category	IDPs (outcome)	Number of SNPs	*β* (IVW)	SE (IVW)	*p*-value (IVW)	*p*-FDR
rfMRI connectivity	rfMRI connectivity (ICA25 edge 203)	42	0.011	0.005	0.034	0.049
	rfMRI connectivity (ICA100 edge 16)	42	0.012	0.005	0.007	0.041
	rfMRI connectivity (ICA100 edge 136)	42	0.015	0.005	0.002	0.038
	rfMRI connectivity (ICA100 edge 416)	42	0.011	0.005	0.0380	0.050
	rfMRI connectivity (ICA100 edge 641)	42	0.012	0.005	0.018	0.048
	rfMRI connectivity (ICA100 edge 651)	42	0.011	0.005	0.042	0.048
	rfMRI connectivity (ICA100 edge 838)	42	0.009	0.005	0.0400	0.048
	rfMRI connectivity (ICA100 edge 857)	42	0.010	0.005	0.031	0.049
	rfMRI connectivity (ICA100 edge 1,426)	42	0.011	0.005	0.020	0.045

### WM tract diffusivity, MO and OD

As shown in Table 4. These significant outcome of brain white matter (WM) tract diffusivity, mode of anisotropy (MO) and orientation dispersion index (OD) were obtained by diffusion-weighted magnetic resonance imaging (dMRI). SLE associated with weighted-mean L3 in tract right acoustic radiation (*β* = 0.012, FDR-adjusted = 0.045). Mean L1 in right anterior corona radiata (*β* = 0.014, FDR-adjusted = 0.038) and left superior cerebellar peduncle (*β* = 0.011, FDR-adjusted = 0.048) on FA (fractional anisotropy) skeleton were affected by SLE. Mean L3 in left medial lemniscus (*β* = 0.010, FDR-adjusted = 0.048), left posterior limb of internal capsule (*β* = 0.012, FDR-adjusted = 0.049) and right posterior limb of internal capsule (*β* = 0.005, FDR-adjusted = 0.033) on FA skeleton were also affected by SLE. Similar results were observed in mean L2 in pontine crossing tract (*β* = 0.011, *p* = 0.050) and mean diffusivity (MD) in left superior cerebellar peduncle (*β* = 0.014, *p* = 0.037). SLE also had a significant effect on mean MO in fornix on FA skeleton (*β* = 0.010, FDR-adjusted = 0.048) and weighted-mean OD in tract left inferior longitudinal fasciculus (*β* = 0.012, FDR-adjusted = 0.046).

**Table 4:** MR results of causal effects between systemic lupus erythematosus and imaging-derived phenotypes (IDPs) in white matter (WM) tract diffusivity, MO and OD.

IDP category	IDPs (outcome)	Description	Number of SNPs	*β* (IVW)	SE (IVW)	*p*-value (IVW)	*p*-FDR
WM tractdiffusivity	IDP_dMRI_ProbtrackX_L3_ar_r	Weighted-mean L3 in tract right acoustic radiation (from dMRI data)	42	0.013	0.006	0.026	0.045
	IDP_dMRI_TBSS_L1_Anterior_corona radiata_R	Mean L1 in anterior corona radiata (right) on FA (fractional anisotropy) skeleton (from dMRI data)	42	0.014	0.005	0.007	0.038
	IDP_dMRI_TBSS_L1_Superior_cerebellar_peduncle_L	Mean L1 in superior cerebellar peduncle (left) on FA (fractional anisotropy) skeleton (from dMRI data)	41	0.011	0.005	0.040	0.048
	IDP_dMRI_TBSS_L2_External_capsule_L	Mean L2 in pontine crossing tract on FA (fractional anisotropy) skeleton (from dMRI data)	42	0.011	0.005	0.033	0.050
	IDP_dMRI_TBSS_L3_Medial_lemniscus_L	Mean L3 in medial lemniscus (left) on FA (fractional anisotropy) skeleton (from dMRI data)	41	0.010	0.005	0.041	0.048
	IDP_dMRI_TBSS_L3_Posterior_limb_of_internal_capsule_L	Mean L3 in posterior limb of internal capsule (left) on FA (fractional anisotropy) skeleton (from dMRI data)	42	0.012	0.005	0.015	0.049
	IDP_dMRI_TBSS_L3_Posterior_limb_of_internal_capsule_R	Mean L3 in posterior limb of internal capsule (right) on FA (fractional anisotropy) skeleton (from dMRI data)	42	0.015	0.005	0.003	0.033
	IDP_dMRI_TBSS_MD_Superior_cerebellar_peduncle_L	Mean diffusivity (MD) in superior cerebellar peduncle (left) on FA (fractional anisotropy) skeleton (from dMRI data)	40	0.014	0.005	0.007	0.037
WM tract MO	IDP_dMRI_TBSS_MO_Fornix	Mean MO (diffusion tensor mode) in fornix on FA (fractional anisotropy) skeleton (from dMRI data)	42	0.010	0.005	0.047	0.048
WM tract OD	IDP_dMRI_ProbtrackX_OD_ilf_l	Weighted-mean OD (orientation dispersion index) in tract left inferior longitudinal fasciculus (from dMRI data)	41	0.012	0.005	0.013	0.046

The results of Cochran’s IVW *Q* test showed no significant heterogeneity of these IVs. In addition, MR-PRESSO analysis find that the global test of mean intensity of caudate in the left hemisphere generated by subcortical volumetric segmentation (aseg), mean intensity of amygdala in the right hemisphere generated by subcortical volumetric segmentation (aseg), rfMRI connectivity (ICA100 edge 638), weighted-mean L3 in tract right acoustic radiation (from dMRI data), and mean L3 in fornix cres + stria terminalis (right) on FA (fractional anisotropy) skeleton (from dMRI data) have horizontal pleiotropy (globle test <0.05), while further analysis did not find any significant outliers. MR-Egger intercepts of other associations were close to zero, suggesting that no significant pleiotropy was detected. And leave-one-out analyses showed that no single SNP drove the causal estimates.

## Discussion

While previous attempts have been made to identify the link between SLE and brain IDP through observational studies, in our study, we identified a number of brain imaging regions associated with SLE that may explain the neuropsychiatric symptoms in SLE patients using genetic causal inference methods. In the present study, we performed two-sample MR analyses to systematically investigate the causal associations between SLE and 3,935 IDPs. The results reflect the association of SLE with patterns of structural and functional brain regions. Some of these structures, including cortical area, white matter regions, anterior corona radiata, and posterior limb of internal capsule, may represent the target brain regions a where SLE acts on cognitive and emotional function.

Neuropsychiatric SLE (NPSLE) comprises the neurologic and psychiatric syndromes observed in patients with SLE in which other causes have been excluded (18). Patients with NPSLE have diverse clinical manifestations, such as mood changes and cognitive problems, symptom severity can vary and objective signs of neuropsychiatric involvement can go undetected (4). The pathogenesis of NPSLE is multifactorial with possible causes including disruption of the blood–brain barrier by intrathecal pro-inflammatory cytokines, neuronal autoantibodies, microangiopathy, chronic diffuse ischemia, thromboembolism and atherosclerosis (19). While NPSLE manifestations usually occur early in the course of SLE, it remains one of the hardest to diagnose and still a major therapeutic challenge (20).

Advances in neuroimaging research suggest that measures of brain function and structure may represent neuropsychiatric impairment. More recent structural studies have used surface-based morphometry, which decomposes cortical volume into its primary components: cortical surface area and thickness (21). These components have distinct evolutionary origins and are regulated by different genetic and cellular processes (22, 23). There is a geometric relationship between global cortical volume, local cortical thickness and surface area (24). ENIGMA (Enhancing Neuro Imaging Genetics through Meta-Analysis) Major Depressive Disorder Working Group identified extensive changes in cortical structure in patients with MDD compared to controls by analysing structural T1-weighted brain magnetic resonance imaging (MRI) scans (25). A few number of observational studies indicated that cortical imaging abnormalities correlated with schizophrenia (26, 27), chronic headache (28), and cognitive dysfunction (29). Li et al. (30) found extensive and obvious reduction in cortical thickness and abnormal topological organization of structural covariance networks (SCNs) in non-NPSLE patients by using MRI-based surface morphometry (SBM) and region of interest (ROI) method. As our findings suggest, SLE affects many cortical structural-functional areas and hemispheric volumes, especially the posterior cingulate. The posterior cingulate cortex is a highly connected and metabolically active brain region (31). Recent studies suggest it has an important cognitive role. However, other evidence suggests that the region may play a direct role in regulating the focus of attention (32). In addition, its activity varies with arousal state and its interactions with other brain networks may be important for conscious awareness (32). Functional neuroimaging studies showed that the posterior cingulate abnormalities were observed in a range of neurological and psychiatric disorders including Alzheimer’s disease, schizophrenia, autism, depression and attention deficit hyperactivity disorder, as well as ageing (33). In SLE patients, posterior cingulate resting state (RS) functional connectivity (FC) is significantly altered in cognitive and psychiatric networks and is involved in the pathophysiology of neuropsychiatric symptoms (34).

The pars triangularis, located within Broca’s area, has been implicated in various aspects of linguistic functioning. More specifically, it has been implicated inverbal working memory, lexical decision making and semantic processing, syntactic processing, phonological decoding and word identification in functional neuroimaging studies (50). There was a study indicated that children with dyslexia have smaller pars triangulari sizes when using manual tracing compared to controls (35). In bipolar disorders, inferior frontal gyrus volume, especially pars triangularis volume were significantly differed from the controls (36). The volume of PuA are included in the thalamus (37). While the thalamus is central to brain functions ranging from primary sensory processing to higher-order cognition. Structural deficits in thalamic association nuclei such as the PuA and mediodorsal nuclei have previously been implicated in neurodegenerative and psychiatric disorders such as multiple sclerosis and schizophrenia (38). Previous study have reported the relationship between thalamus alterations and SLE, which may affect the nervous system, causing NPSLE (39, 40). However, few study focus on the volume of PuA. Significant results of pars triangularis and PuA affected by SLE may explain some of the NPSLE symptoms.

As mentioned above, SLE was causally associated with rfMRI IDPs in our study, accounting for almost half of all associations. rfMRI is a method that can be applied with different spatial (millimeters) and temporal (seconds) resolutions, as well as use of graph theory to quantify functional connectivity from spontaneous fluctuations in brain activity (41, 42). Functional connectivity mapping of rfMRI is a promising tool for measuring macro-scale networks in the human brain and studying *in vivo* neural mechanisms of neurologic and psychiatric disorders. In an observational study, functional connectivity also correlated with mental disorder, which weaker in patients with MDD within and between all brain networks compared with healthy controls (43). Functional connectivity abnormalities have also been described both in NPSLE-patients and SLE-patients without neuropsychiatric involvement (non-NPSLE-patients) (44, 45).

dMRI have also been used to assessing properties of WM in brain. dMRI is a popular noninvasive tool which may explore the topographical organization of structural and functional brain connectivity. The FA, mean diffusivity (MD), diffusion tensor mode (MO), intracellular volume fraction (ICVF), isotropic or free water volume fraction (ISOVF), and orientation dispersion index (OD) indices obtained from dMRI after post-processing were used to measure structural connectivity. These indices have been reported to assess WM fiber damage in Alzheimer disease patients. In patients with SLE, its alteration correlated with neuropsychiatric involvement and autoantibody profiles (46). Recent studies have found that SLE patients with or without neuropsychiatric symptoms have significantly lower FA values and higher MD values in multiple WM regions compared to healthy control, and subclinical microstructural changes have also been observed in either regional areas or the entire brain by dMRI (47), which consistent with our results. FA and OD of white matter are associated with depression, anxiety, PTSD, psychosis and somatisation symptoms (48). Previous Mendelian randomization analyses also support causal relationships between brain imaging-derived phenotypes and risk of psychiatric disorders (49). Similarly, our MR analyses identified IDPs that can be used to assess neuronal changes and reflect axonal properties (e.g., FA, MD, MO, and OD in white matter), which may indicate that the psychiatric symptoms of NPSLE have similarities with psychiatric disorders such as schizophrenia.

In addition, our study identified a number of brain structural and functional regions affected by SLE that had not been identified in previous observational studies. These regions are thought to be associated with psychiatric disorders and cognitive changes in previous studies. All these associations may suggest the pathogenesis of neuropsychiatric symptoms in patients with SLE and be useful for the diagnosis and assessment of SLE.

There are limitations to our study. First, although most participants in the GWAS summary statistics for SLE data were of European descent, there may still be interference from population stratification, and the results of this study may not be entirely applicable to subjects of non-European descent. Future MR studies on the causal association between SLE and IDPs could be considered in diverse European and non-European populations for better generalizability. Second, because summary statistics rather than raw data were used in the analysis, it was not possible to perform subgroup analyses, such as distinguishing NPSLE and non-NPSLE. Further studies are warranted to research the association between NPSLE and non-NPSLE with brain IDPs.

## Conclusion

In conclusion, we conducted two-sample MR analyses to systematically estimate the underlying causal relationships between SLE and neuroimaging phenotypes using large-scale GWAS data. The findings revealed strong genetic evidence for causal links between SLE and neuroimaging phenotypes. This will contribute to better prediction and intervention at the brain-imaging level for the risk of NPSLE.

## Data Availability

The original contributions presented in the study are included in the article/Supplementary material, further inquiries can be directed to the corresponding author.

## Footnotes

^1^ https://www.ebi.ac.uk/gwas/

^2^ https://biobank.ctsu.ox.ac.uk/crystal/crystal/docs/brain_mri.pdf

^3^ https://open.win.ox.ac.uk/ukbiobank/big40/
